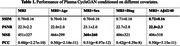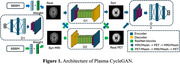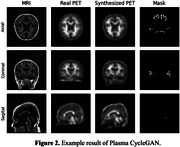# Plasma CycleGAN: Integrating Blood‐based Biomarkers for Cross‐modality Translation from MRI to PET

**DOI:** 10.1002/alz.095616

**Published:** 2025-01-09

**Authors:** Yanxi Chen, Yi Su, Yu Fu, Kewei Chen, David Weidman, Richard J. Caselli, Eric M. Reiman, Yalin Wang

**Affiliations:** ^1^ Arizona State University, Tempe, AZ USA; ^2^ Banner Alzheimer’s Institute, Phoenix, AZ USA; ^3^ Zhejiang University, Hangzhou, Zhejiang China; ^4^ Mayo Clinic Arizona, Scottsdale, AZ USA

## Abstract

**Background:**

Cross‐modality translation between MRI and PET presents significant challenges due to the distinct mechanisms underlying those modalities. Currently, the leading method for this translation is CycleGAN. Previous studies have proved a strong correlation between BBBMs and brain amyloid status measured by PET. However, the impact of blood‐based biomarkers (BBBMs) on PET image synthesis has not yet been thoroughly evaluated. In this study, we propose Plasma CycleGAN, a generative model based on CycleGAN to synthesize PET images from MRI, while also incorporating BBBMs and other clinical covariates. We show that Plasma CycleGAN outperforms the state‐of‐the‐art CycleGAN for MRI‐to‐PET translation. Our method is the first to integrate BBBMs in a conditional cross modality translation from MRI to PET.

**Method:**

Our model was built on CycleGAN architecture, while the backbone was improved by integrating BBBMs as additional conditions in the input domain. To be specific, we introduce a masked perturbation in the MRI image, whose voxel‐level magnitudes are learnable parameters in the neural network. The dataset we used to evaluate our model is downloaded from the ADNI database. The cohort contains 160 subjects with matching BBBM, T1‐weighted MRI images, amyloid PET, and clinical features, including age, sex, weights, etc. The synthesized PET images are compared with the ground truth PET images for performance evaluation using traditional metrics like structural similarity index (SSIM), peak‐signal‐to‐noise ratio (PSNR) and mean square error (MSE). In addition, the Pearson correlation coefficients (PCCs) of the masked SUVR ratios were calculated.

**Result:**

We evaluated the baseline CycleGAN model using MRI input, as well as combined inputs of MRI+BBBM and other clinical features. Our model achieved an SSIM of 0.72±0.16 and PSNR of 22.8±2.3 in PET image synthesis task using MRI+ Aβ42/40, both yielding the best results among all input combinations. MRI+sex achieved the best result using MSE measurement. PCCs of all experimental results ranged from 0.44 to 0.5.

**Conclusion:**

We introduce Plasma CycleGAN, the first generative model to integrate BBBMs and clinical features in PET image synthesis from MRI images. In PET image synthesis task, our proposed integrated model outperforms the baseline CycleGAN model.